# Studies on the *In Vitro* Antiproliferative, Antimicrobial, Antioxidant, and Acetylcholinesterase Inhibition Activities Associated with *Chrysanthemum coronarium* Essential Oil

**DOI:** 10.1155/2015/790838

**Published:** 2015-07-28

**Authors:** Sanaa K. Bardaweel, Mohammad M. Hudaib, Khaled A. Tawaha, Rasha M. Bashatwah

**Affiliations:** ^1^Department of Pharmaceutical Sciences, Faculty of Pharmacy, The University of Jordan, Amman 11942, Jordan; ^2^College of Pharmacy, Taibah University, Al Madinah Al Monawarrah 41447, Saudi Arabia

## Abstract

The essential oil of the Jordanian *Chrysanthemum coronarium* L. (garland) was isolated by hydrodistillation from dried flowerheads material. The oil was essayed for its *in vitro* scavenging activity using the 1,1-diphenyl-2-picrylhydrazyl (DPPH) method. The results demonstrate that the oil exhibits moderate radical scavenging activity relative to the strong antioxidant ascorbic acid. In addition, cholinesterase inhibitory activity of *C. coronarium* essential oil was evaluated for the first time. Applying Ellman's colorimetric method, interesting cholinesterase inhibitory activity, which is not dose dependent, was evident for the oil. Furthermore, antimicrobial activities of the oil against both Gram-negative and Gram-positive bacteria were evaluated. While it fails to inhibit Gram-negative bacteria growth, the antibacterial effects demonstrated by the oil were more pronounced against the Gram-positive strains. Moreover, the examined oil was assessed for its *in vitro* antiproliferative properties where it demonstrated variable activities towards different human cancer cell lines, of which the colon cancer was the most sensitive to the oil treatment.

## 1. Introduction

The genus* Chrysanthemum* belongs to the Asteraceae family and consists of nearly 300 species [1].* Chrysanthemum* has been thoroughly investigated for its species' biological activities and chemical compositions. For example,* C. morifolium*, which is widely used in China as a dietary supplement [2, 3], has been shown to exhibit antihepatotoxic and antigenotoxic effects [4]. In addition,* C. indicum*, a traditional herb that is widely used in Korea and Japan, has been found to possess anti-inflammatory, immunomodulatory, humoral and cellular, and mononuclear phagocytic activities [5, 6]. Also, the flowers of* C. cinerariaefolium* and* C. macrotum* were proven to have insecticidal and herbicide activities [1, 7].

*Chrysanthemum coronarium* is an annual herbaceous weed that is broadly spread in the Mediterranean region, Japan, China, and The Philippines and occasionally introduced and naturalized elsewhere [8, 9]. In Jordan, the plant is commonly known as crown daisy or garland in English and as “Besbas” or “Gassoum” in Arabic.* Chrysanthemum coronarium* is an ornamental plant which is appreciated as a Chinese vegetable. The nutritional composition of* C. coronarium* has been described by Willis et al. [10].* Chrysanthemum coronarium* is frequently used in food industry in Asian countries, such as Japan and China, as antioxidant, antibacterial, and antifungal food additive [11]. In Jordan, the flowers of the plant are used as plasters for dermal diseases and as a vermifuge [12].

The diversity in biological activities of the plant was attributed to the chemical heterogeneity of its composition [11, 13]. Secondary metabolites, such as essential oils and flavonoids, were reported to considerably contribute to some of the biological activities associated with the plant [14]. Interestingly, the chemical composition and the phenolic profile of* C. coronarium* essential oil are noticeably inclined to quantitative and qualitative changes owing to genotype, geographic origin, and environmental factors [15]. For instance, the main components of the essential oil from Spanish samples were camphor (29.2%), *α*-pinene (14.8%), lyratyl acetate (9.8%), and *β*-pinene (8.5%) [14]. On the other hand, the essential oil composition of* C. coronarium* flowerheads from Tunisian origin was found to be* cis*-chrysanthenyl acetate (21.82%),* trans*-chrysanthenyl acetate (12.78%), (E)-*β*-farnesene (8.97%), germacrene-D (8.92%), and camphor (6.03%) [16]. Nonetheless, the principal oil component of* C. coronarium* flowerheads from Jordan was found to be camphor (17.5%) whereas santolina triene (4.3%), neoiso-3-thujanol (5.6%),* cis*-chrysanthenyl acetate (10.8%), perilla aldehyde (11.7%), isoitalicene (4.7%), and phenylpropyl butanoate (4.9%) were the major components of the oil [17]. Collectively, Tawaha and Hudaib [17] reported that the chemical composition of the Jordanian specimen was nearly similar, particularly qualitatively, in terms of the main components to those reported in the literature, except in the relative quantities of the principal and major components. Interestingly, distinctive abundance of the phenolic fraction in the Jordanian garland oil, notably phenylpropyl butanoate, was also reported [17].

Despite the aforementioned knowledge on the chemistry of essential oil from the Jordanian* C. coronarium*, the biological activities associated with the oil have not been yet evaluated. Therefore, the present study was designed to investigate the potential biological activities the* C. coronarium* oil may exhibit.

## 2. Materials and Methods

### 2.1. Plant Material

The flowerheads have been separated from* C. coronarium* plants collected from Houfa (Irbid governorate, Jordan) during the full flowering stage of the plant. The plant material was botanically identified by Professor Khaled Tawaha (Department of Pharmaceutical Sciences, Faculty of Pharmacy, The University of Jordan). A voucher specimen (ID: C.CorHud-04-14) of the collected plants was deposited in the research laboratory of the Department of Pharmaceutical Sciences, Faculty of Pharmacy, UJ.

### 2.2. Essential Oil Extraction

The plant materials were air-dried at room temperature (20 ± 2°C) for one week, ground to about 0.5 mm particle size (30–35 mesh), and submitted to hydrodistillation for 4 h using a Clevenger-type apparatus (JSGW, India). The oil obtained was dried over anhydrous sodium sulphate (BDH Analar, UK) and stored in amber and air-tight sealed vials at 4°C until analyzed. The yield of oil was calculated as percentage volume per weight (% v/w) of the dried plant material.

### 2.3. Antiradical Scavenging Activity

The electron donation ability of the hydrodistilled essential oil was measured as a decrease in the absorbance of methanol solution of 1,1-diphenyl-2-picrylhydrazyl (DPPH) and the bleaching of the purple-colored solution. In brief, a stock solution of DPPH (0.1 mM) was prepared in methanol and different concentrations of the essential oil (30 *µ*L) were added (50–300 *μ*g/mL) to 3 mL of freshly prepared methanol solution of DPPH [18]. After incubation at room temperature for 30 min, the pale pink color developed was measured spectrophotometrically at 517 nm and compared with the standard ascorbic acid. The radical scavenging activity was calculated by the DPPH inhibition percentage as follows:

(1) Antiradical activity  %=Control Absorbance−Sample AbsorbanceControl Absorbance·100.

### 2.4. Acetylcholinesterase Inhibition

Inhibition of acetylcholinesterase (AChE) activity was determined using Ellman's colorimetric method as modified by Eldeen et al. [19]. Briefly, 25 *μ*L of 15 mM ATCI in water, 125 *μ*L of 3 mM DTNB in Buffer C (50 mM Tris-HCl, pH 8, containing 0.1 M NaCl and 0.02 M MgCl_2_·6H_2_O), 50 *μ*L of Buffer B (50 mM, pH 8, containing 0.1% bovine serum albumin), and 25 *μ*L of the examined essential oil were all placed in reaction wells. Absorbance was measured spectrophotometrically using microplate reader at 405 nm every 45 s, three times successively. Following, AChE (0.2 U/mL) was added to the reaction mixture and the absorbance was measured five times consecutively every 45 s. Galantamine was used as the positive control indicative of AChE inhibition. To correct for any increase in absorbance due to the substrate spontaneous hydrolysis, the absorbance before adding the enzyme was subtracted from the absorbance after adding the enzyme. The percentage inhibition was calculated using the following formula:

(2) $$\begin{array}{c} \text{Inhibition}\left(\;\%\;\right)=1-\left(\;\frac{A_{\text{sample}}}{A_{\text{control}}}\;\right)\cdot 100, \end{array}$$

where *A*_sample_ is the absorbance of the sample and *A*_control_ is the absorbance of the blank [methanol in Buffer A (50 mM Tris-HCl, pH 8)].

### 2.5. Antimicrobial Activity

The paper-disk diffusion method was used for the evaluation of the antibacterial activity of the essential oil. In this assay,* Bacillus subtilis* ATCC 11562,* Staphylococcus aureus* ATCC 6538,* Staphylococcus epidermidis* ATCC 12228,* Escherichia coli* ATCC 29425, and* Pseudomonas aeruginosa* ATCC 11921 were tested. Briefly, a suspension of the tested microorganisms (10^6^ CFU/mL) was spread on the Mueller-Hinton media plates. Sterile filter paper disks (6 mm in diameter) were soaked with 20 *μ*L of the oil and placed on the inoculated plates. After incubation at 37°C for 24 h, the diameters of inhibition zones (mm) were measured. When the diameter of the inhibitory zone is equal to 6 mm, the oil tested was considered as not active. Norfloxacin (1 mg/mL) was used as positive reference standard to determine the sensitivity of microbial strains tested.

### 2.6. Antiproliferative Activity

To study changes in mitochondrial/nonmitochondrial dehydrogenase activity and, thus, estimate the essential oils induced antiproliferative activity, the MTT [3-(4,5-dimethylthiazol-2-yl)-2,5-diphenyltetrazolium bromide] (Sigma Chemical Co., USA) assay was used. Cells were seeded on 96-well plates and incubated with different concentrations of the essential oil (5–200 *μ*g/mL). When the incubation period finished, the supernatant was removed and MTT solution (0.5 mg/mL) was added to each well. The formed dark blue formazan crystals were solubilized in DMSO, and the absorbance was measured at 570 nm using a microplate reader. Cell survival was assessed by comparing the absorbance values obtained from treated and untreated cells. As a positive control, vincristine sulphate (Sigma) was used at a concentration of 100 nM. In addition, a negative control, control wells without essential oils treatment, was employed and prepared under the same experimental conditions. The survival curves for cell lines treated with the essential oil were obtained by plotting the relationships between surviving fractions and essential oil concentrations. The curves were fitted using linear equation, and the 50% inhibiting concentration (LD_50_) was determined. The LD_50_ value was defined as the concentration of essential oil that results in a 50% reduction of the surviving fraction.

### 2.7. Statistical Analysis

All results were computed and expressed as mean ± standard deviation (SD) from three determinations performed in triplicate (*n* = 9). Statistical analysis was performed using SPSS software (version 17.0) with analysis of variance (one-way ANOVA) and post hoc test Dunnett's T3 that were used to contrast the significant difference between the groups. A *p* value of <0.05 was considered as statistically significant.

## 3. Results and Discussion

**Figure 1:**
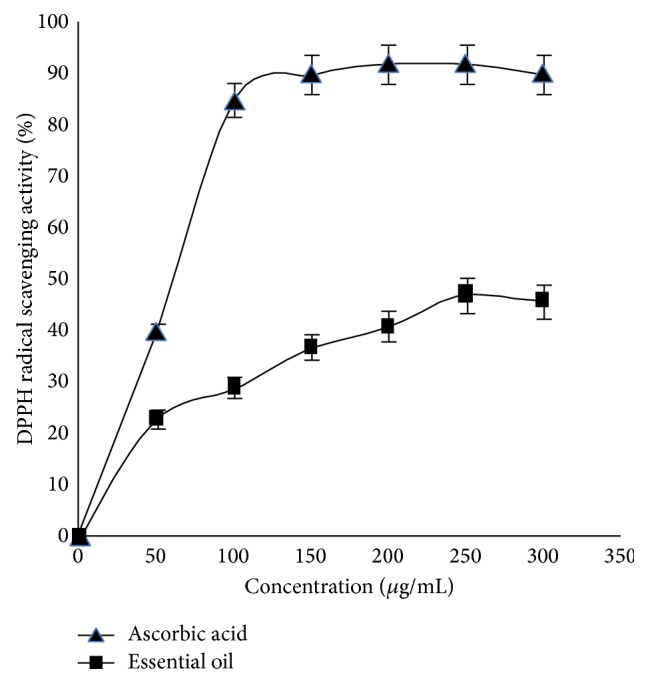
DPPH radical scavenging activity of* Chrysanthemum coronarium* essential oil and the positive control ascorbic acid.

The efficiency of the examined essential oil free radical DPPH scavenging is illustrated in Figure 1. As depicted, the essential oil demonstrates moderate radical scavenging activity relative to the strong antioxidant ascorbic acid (*p* value < 0.05). At the maximum concentration used of the oil, stable violet DPPH radical was reduced to the yellow-colored DPPH-H form with only about half-capacity of the ascorbic acid. This moderate DPPH scavenging activity of the oil may be partially attributable to its poor solubility in a polar medium and that the observed antioxidant activity is due to the low oil fraction that reached the reaction solution. Consistent with previous literature reports, the majority of essential oils examined for their free radical scavenging activities fail to demonstrate immense activity relative to water soluble strong antioxidants [18, 20, 21].

**Figure 2:**
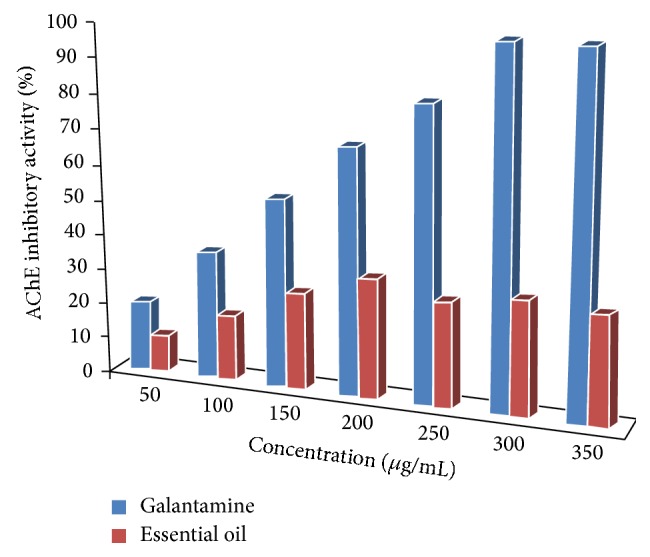
AChE inhibitory activity of* Chrysanthemum coronarium* essential oil and the positive control galantamine.

Several essential oils are reputed to enhance memory. Of the most essential oils tested for improving study skills and test taking ability are the Lemon and Rosemary essential oils [22]. The most acceptable theory for their memory-enhancing/antidementia activities is their ability to enhance the cholinergic function by inhibiting cholinesterase [23]. The results of the AChE inhibitory activities of the essential oil relative to the positive control, galantamine, are illustrated in Figure 2. As shown, the essential oil exhibited some level of inhibitory activity against AChE. Relative to the dose-dependent response of the positive control galantamine, the essential oil does not appear to inhibit AChE in a dose-dependent manner, which makes it difficult to estimate the IC_50_ value for the essential oil. It is speculated that the anticholinesterase activity of the major monoterpenoid constituent (camphor) is responsible for the observed cholinesterase inhibition activity of the oil. Interestingly, camphor was found to be uncompetitive reversible inhibitor of the cholinesterase with poor IC_50_ value of >10 mM [24]. Nonetheless, Miyazawa et al. [25] reported that the anticholinesterase activity exhibited by essential oils is usually more potent than that of their major constituents. Although there is a possibility of synergistic activity taking place between the oils major components enhancing the ability of the oil to deactivate AChE, it is also proposed that other trivial unidentified constituents of the oil might be solely responsible for the observed inhibition.

Evidence has begun to accumulate supporting the role of oxidative stress to initiate and accelerate several age-related neurodegenerative diseases [24]. Positive outcomes of antioxidants to limit neuronal death occurring in the pathophysiology of these disorders have been clearly demonstrated in numerous studies [26]. The antioxidant potential of a chemical can be related to its radical scavenging ability. Our results indicate that* C. coronarium* essential oil showed a propensity to quench the free radicals in the DPPH assay indicating the potential of its antioxidant activity.

In addition, several plant species were found to contain AChE inhibitory alkaloids, such as galantamine, which was originally discovered in the bulbs of* Galanthus nivalis*, and currently are used for the treatment of mild to moderate Alzheimer's disease [27]. Interestingly, new naturally occurring cholinesterase inhibitors continue to be identified in numerous plant species. Although the exact identity of the chemical compounds responsible for the cholinesterase inhibition is quite challenging and has not been fully characterized, several monoterpenoids, occurring in essential oils of related plants, were suggested to potentially inhibit cholinesterase [24]. Based on the results of this study, it is highly suggested that the essential oil obtained from the flowerheads of* C. coronarium* must be further investigated for possible beneficial effects in neurodegenerative diseases, such as Alzheimer's disease, utilizing its moderate antioxidant and weak cholinesterase inhibition activities.

The antimicrobial activity of the essential oil of* C. coronarium* examined in the present study was qualitatively evaluated by the presence or absence of inhibition zones and zone diameter. The results given in Table 1 demonstrate that the essential oil exhibits antimicrobial activity against Gram-positive bacteria while it fails to inhibit the growth of Gram-negative bacteria. Generally, these results are consistent with previous literature findings that Gram-positive bacteria are more sensitive than Gram-negative towards essential oils treatments [18, 28]. The occurrence of a very prohibitive outer membrane barrier in Gram-negative bacteria is thought to be the primary reason for the intrinsic resistance possessed by Gram-negative bacteria towards antibacterial treatments.

**Figure 3:**
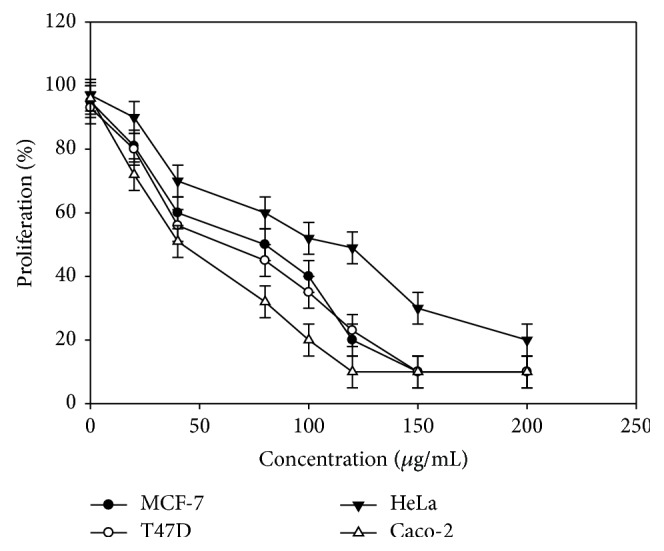
Antiproliferative activities of* Chrysanthemum coronarium* essential oil against four human cancer cell lines. Exposure time 48 h. Values are expressed as mean ± SD of three experiments.

Furthermore, the examined oil was assessed for its* in vitro* cytotoxic properties on four human cancer cell lines, including human breast adenocarcinoma MCF-7 cell line, the human ductal breast epithelial tumor T47D cell line, the human colon adenocarcinoma Caco-2 cell line, and the human epithelial carcinoma HeLa cell line. Cytotoxic effects of the oil on the growth of the studied cell lines are presented in Table 2, which shows the concentration of the oil at which cancer cells growth was inhibited by 50% (LD_50_). According to LD_50_ values, the oil exhibited potent* in vitro* antiproliferative properties on the cell lines under investigation. The different responses to oil treatment demonstrated by the different cell types examined can be attributed to the versatility of the oil composition and the different pathways through which the oil components may interact with different cell types (Figure 3) [18, 28].

## 4. Conclusion

To the best of our knowledge, this is the first comprehensive study providing new insights into the biological activities of the Jordanian garland* Chrysanthemum coronarium* essential oil. The essential oil appears to be moderately active as a radical scavenger with weak acetylcholinesterase inhibition potential. In addition, satisfying antibacterial activities were exhibited by the oil against Gram-positive strains relative to its poor anti-Gram-negative potential. The antiproliferative activities of the oil indicate that* C. coronarium* essential oil might be effective for growth inhibition of various tumor cell lines with LD_50_ values ranging from 43 to 110 *µ*g/mL.

## Figures and Tables

**Table 1:** Antibacterial activity of *Chrysanthemum coronarium* essential oil measured by zone of inhibition (mm). Results represent the means of three independent readings.

Microorganism	*Essential oil *	Norfloxacin
*Bacillus subtilis *	19	17
*Staphylococcus aureus *	20	14
*Staphylococcus epidermidis *	18	21
*Escherichia coli *	9	18
*Pseudomonas aeruginosa *	12	27

**Table 2:** Evaluation of antiproliferative activities of *Chrysanthemum coronarium* essential oil by MTT assay in cell lines after 48 h. The presented LD_50_ values are expressed as *μ*g/mL ± SD and correspond to the means of three independent readings.

Cell line	LD_50_ (*μ*g/mL)
Human ductal breast epithelial tumor* T47D *	75 ± 5
Human breast adenocarcinoma* MCF-7 *	82 ± 8
Human colon adenocarcinoma* Caco-2 *	43 ± 6
Human epithelial carcinoma* HeLa *	110 ± 8
